# Normal organ dosimetry for thyroid cancer patients treated with radioiodine as part of the multi-centre multi-national Horizon 2020 MEDIRAD project

**DOI:** 10.1007/s00259-023-06295-0

**Published:** 2023-06-10

**Authors:** Jan Taprogge, Alex Vergara-Gil, Francesca Leek, Carla Abreu, Lenka Vávrová, Lily Carnegie-Peake, Sarah Schumann, Uta Eberlein, Michael Lassmann, Tino Schurrat, Markus Luster, Frederik A. Verburg, Delphine Vallot, Lavinia Vija, Frédéric Courbon, Kate Newbold, Manuel Bardiès, Glenn Flux

**Affiliations:** 1 National Radiotherapy Trials Quality Assurance (RTTQA) Group, Joint Department of Physics, Royal Marsden NHSFT, Downs Road, Sutton, SM2 5PT UK; 2 The Institute of Cancer Research, 123 Old Brompton Road, London, SW7 3RP UK; 3 Centre de Recherches en Cancérologie de Toulouse, UMR 1037, INSERM Université Paul Sabatier, Toulouse, France; 4 Joint Department of Physics, Royal Marsden NHSFT, Downs Road, Sutton, SM2 5PT UK; 5 Department of Nuclear Medicine, University of Würzburg, Oberdürrbacher Str. 6, 97080 Würzburg, Germany; 6 Department of Nuclear Medicine, Philipps-University Marburg, Baldingerstrasse, 35043 Marburg, Germany; 7 Department of Radiology and Nuclear Medicine, Erasmus Medical Center, Doctor Molewaterplein 40, 3015 GD Rotterdam, Netherlands; 8 IUCT Oncopole, Av. Irène Joliot-Curie, 31100 Toulouse, France; 9 Thyroid Unit, Royal Marsden NHSFT, Downs Road, Sutton, SM2 5PT UK; 10 Institut de Recherches en Cancérologie de Montpellier, UMR 1194, INSERM Université de Montpellier, 34298 Montpellier, France

**Keywords:** Multicentre study, Dosimetry, NaI, Thyroid cancer

## Abstract

**Purpose:**

Dosimetry is rarely performed for the treatment of differentiated thyroid cancer patients with Na[^131^I]I (radioiodine), and information regarding absorbed doses delivered is limited. Collection of dosimetry data in a multi-centre setting requires standardised quantitative imaging and dosimetry. A multi-national, multi-centre clinical study was performed to assess absorbed doses delivered to normal organs for differentiated thyroid cancer patients treated with Na[^131^I]I.

**Methods:**

Patients were enrolled in four centres and administered fixed activities of 1.1 or 3.7 GBq of Na[^131^I]I using rhTSH stimulation or under thyroid hormone withdrawal according to local protocols. Patients were imaged using SPECT(/CT) at variable imaging time-points following standardised acquisition and reconstruction protocols. Whole-body retention data were collected. Dosimetry for normal organs was performed at two dosimetry centres and results collated.

**Results:**

One hundred and five patients were recruited. Median absorbed doses per unit administered activity of 0.44, 0.14, 0.05 and 0.16 mGy/MBq were determined for the salivary glands of patients treated at centre 1, 2, 3 and 4, respectively. Median whole-body absorbed doses for 1.1 and 3.7 GBq were 0.05 Gy and 0.16 Gy, respectively. Median whole-body absorbed doses per unit administered activity of 0.04, 0.05, 0.04 and 0.04 mGy/MBq were calculated for centre 1, 2, 3 and 4, respectively.

**Conclusions:**

A wide range of normal organ doses were observed for differentiated thyroid cancer patients treated with Na[^131^I]I, highlighting the necessity for individualised dosimetry. The results show that data may be collated from multiple centres if minimum standards for the acquisition and dosimetry protocols can be achieved.

**Supplementary Information:**

The online version contains supplementary material available at 10.1007/s00259-023-06295-0.

## Background

The treatment of differentiated thyroid cancer (DTC) with Na[^131^I]I (radioiodine) following thyroidectomy remains subject to debate [1]. Treatment approaches vary from not administering Na[^131^I]I [2] to the possibility of dosimetry-based administrations [3]. Results of the ESTIMABL2 trial [4] showed that treatment strategies for patients with low-risk DTC not administered Na[^131^I]I were non-inferior to treatment with Na[^131^I]I with respect to functional, structural and biologic events at 36 months. The randomised trials HiLo [5, 6] and ESTIMABL1 [7] showed no difference between 1.1 and 3.7 GBq with respect to post-ablation success at 6–9 months and recurrence rates. Although these studies were performed with empirical activities, several studies have hypothesised that ablation success would be more closely related to the absorbed doses delivered than to the administered amount of activity [8–11].

An optimised treatment strategy would ideally be based on the risk-to-benefit ratio for individual patients and established absorbed dose–response relationships and the potential risks of low irradiations of healthy organs. Possible side effects from Na[^131^I]I treatment are salivary gland disorders [12, 13] and secondary primary malignancies [14–16] although incidence rates vary significantly between studies. Retrospective epidemiological studies have presented contradicting results and have seldom included dosimetry of healthy organs.

Prospective multi-national multi-centre clinical or epidemiological studies that incorporate standardised quantitative imaging and dosimetry networks are necessary to overcome the limitation of small number of patients treated at individual centres [17, 18]. A study within the EU Horizon MEDIRAD project [19] performed a multi-centre prospective clinical study to assess the absorbed doses delivered to healthy organs and target volumes for DTC patients treated with Na[^131^I]I. In addition, bio-kinetic models were revised and developed for this patient population [20] and the DNA damage and repair in peripheral blood mononuclear cells were assessed [21].

We report here on an observational study employing standardised quantitative imaging and dosimetry. We present the range of absorbed doses delivered to healthy organs. We also identify and address issues when full standardisation cannot be achieved.

## Methods

A multi-centre multi-national prospective observational study was performed within the EU MEDIRAD programme [19]. Patients were recruited onto the study within each participating country with study inclusion criteria and trial endpoints aligned between the centres. The primary endpoint was to establish the range of absorbed doses to target tissues and healthy organs from Na[^131^I]I. Three separate clinical trials, one in each participating country, were approved by the respective national and institutional review boards (see Supplementary Table 1). All patients provided written informed consent prior to registration.

### Quantitative SPECT imaging network

The four participating clinical imaging centres (University Hospital of Marburg (UMR) Germany, centre 1; University Hospital Würzburg (UKW) Germany, centre 2; Institut Universitaire du Cancer de Toulouse (IUCT-O) France, centre 3 and Royal Marsden Hospital (RMH) United Kingdom, centre 4) had been set-up as a European network of centres able to perform standardised quantitative imaging of Na[^131^I]I [17]. Site set-up measurements included assessment of system volume sensitivity to quantify the images and determination of recovery coefficients to account for the apparent loss in activity due to the partial volume effect.

The standardised image acquisition and reconstruction protocols have been reported in a previous publication [17] and are included as Supplementary Tables 2 and 3.

### Patient inclusion criteria

Patients were included in the study if they had histologically proven DTC and a total or staged (hemithyroidectomy followed by completion thyroidectomy) thyroidectomy. Only patients 18 years or older and treated for the first time with radioactive iodine (RAI) were eligible for participation. Patients were excluded from the study if they had a prior diagnostic Na[^131^I]I scan, external beam radiotherapy or systematic chemotherapy within 6 weeks of treatment. No salivary gland stimulation protocols were defined in the clinical trial protocols.

### Data collection and imaging schedule

Additional clinical data required for the dosimetry analysis in this cohort were collected with standardised case report forms (CRFs) in all centres and were transcribed to an electronic CRF (e-CRF) [22]. Imaging data were uploaded onto a central DICOM repository (Kheops) and the Image and Radiation Dose Biobank (IRDBB) [23].

While standardised image acquisition and reconstruction protocols were implemented for the SPECT acquisitions, a flexible imaging schedule was implemented throughout the studies to allow for local differences in imaging system availability, ethics approval and due to COVID-19 restrictions. Patients could be enrolled in the study with a single-photon emission computed-tomography (SPECT) scan between 24 and 96 h post administration of Na[^131^I]I. Up to five optional SPECT scans were collected, where possible, from 6 to 168 h post administration. Patients enrolled with a single or multiple SPECT scans are referred to hereafter as single-time-point and multiple-time-point patients, respectively. A single computed tomography (CT) scan was acquired together with one of the SPECT scans for each patient for attenuation correction and Monte Carlo absorbed dose calculations. Additional CT scans were not acquired due to restrictions imposed in the ethics approval process and concerns raised by patients. One centre had a SPECT-only system for which Chang’s attenuation correction was used in place of CT-based attenuation correction. Reconstruction of scans was performed locally according to the standardised protocol provided in Supplementary Table 3.

Regular whole-body (WB) retention measurements were performed during the patient’s stay in hospital according to local standard of care procedures and the quantified level of radioactivity in the WB was estimated for each time-point. Retention measurements were performed for up to 7 days post administration for centres 1 and 2, while centres 3 and 4 acquired data for up to 4 days due to shorter inpatient stays.

### Dosimetry calculations

Dosimetry calculations were performed by two dosimetry teams. Each independently analysed the data collected at centre 4 for comparison.

#### Dosimetry methodologies for dosimetry team A

Dosimetry team A (DTA, Centre de Recherches en Cancérologie de Toulouse) performed dosimetry calculations from data acquired at centres 2 to 4 using OpenDose3D [24–26], an extension to 3DSlicer [27, 28] developed as part of the OpenDose project [29]. The extension relies on the existing open source architecture of 3DSlicer designed for medical image analysis and includes modules specifically designed for molecular radiotherapy (MRT) dosimetry such as calculation of absorbed dose (rates) from 3D maps of density and cumulated activity (activity) and the integration of time-dependent parameters including activity (to provide cumulated activity or time-integrated activity), or absorbed dose rates (to provide the absorbed dose). SPECT images were registered using rigid deformation in the Elastix module of Slicer3D.

The following organs were segmented using 3DSlicer tools if included in the field-of-view (FOV): neck uptake, lungs (left/right), salivary glands, bones, liver, kidneys (left/right), spleen, urinary bladder and L2–L4. Manual or threshold-based segmentation was performed on functional or anatomical images. Image data were quantified using the system–volume calibration factors determined for each imaging system [17] and activity in each volume-of-interest (VOI) at each time-point was calculated by summing the activity contained in individual voxels in the respective VOI. The integration of activity over time was then performed for each VOI, assuming a mono-exponential decay to determine time-integrated activity coefficients (TIAC). For single-time-point patients (all patients recruited in centre 3 and 12 out of 25 patients recruited in centre 4), the effective half-life derived from whole-body external counting was used for all organs except the neck region where a fixed 68-h effective half-life was used taken from literature for an rhTSH-treated patient population [30]. All single-time-point patients were treated using rhTSH stimulation.

Monte Carlo modelling was performed to derive voxel-based absorbed dose rates for each time-point. A single CT was used for each time-point for both attenuation correction and Monte Carlo simulation using GATEv8.2 [31]. Time integration of the mass averaged absorbed dose rates, the total deposited energy in the VOI divided by the VOI mass, was performed for each VOI, similar to the method described above for the TIAC.

#### Dosimetry methodologies for dosimetry team B

Dosimetry team B (DTB, Royal Marsden Hospital) performed absorbed dose calculations for centres 1 and 4 using in-house dosimetry software developed in 3DSlicer [27, 28]. Images were quantified using system–volume calibration factors determined for each imaging system [17] and the area-under-the-curve was determined using single or multiple time-point fitting as applicable.

For single time-point patients, assumed half-lives of *T*_1/2_ = 9.3 and 8.6 h were used for the parotid and submandibular salivary glands, respectively, which were taken from literature [32]. Salivary glands were segmented using the tools available in 3DSlicer, taking into account the anatomical information from the CT (if available) to determine the volume. Outlining on the SPECT scans was performed either via thresholding (centre 1 where anatomical imaging information was not available) or by copying the CT outline onto the SPECT scans (centre 4) to obtain the activity retention. For thresholding, a fixed threshold of 35% was used, determined from a comparison of anatomical and functional image segmentation in patients of centre 4. The mean absorbed dose to salivary glands was obtained using dose kernel convolution, taking into account the contribution of charged particles to the absorbed dose only.

#### Whole-body dosimetry

WB absorbed doses were estimated from the WB retention measurements. The WB absorbed dose is frequently used as a surrogate for the absorbed dose to the bone marrow [33]. The time integrated activity was obtained from a multi-exponential fit to the data using Solver, a Microsoft Excel add-in programme. The medical internal radiation dose (MIRD) [34] formalism was employed for the calculations using a mass-adjusted (${m}_{p}$, the patient’s weight in kg) S-factor as proposed by Buckley et al. [35]:

1 $$S_{\mathrm{WB}\leftarrow\mathrm{WB}}=1.34\times10^{-4}\times m_p^{-0.921}\mathrm{Gy}\;\mathrm{MBq}^{-1}\mathrm h^{-1}.$$

#### Statistical analysis

The Mann–Whitney test was employed to assess whether WB absorbed doses per unit administered activity were significantly different between patients treated with 1.1 and 3.7 GBq and between rhTSH stimulation and THW, respectively. Furthermore, the Mann–Whitney test was used to assess differences between the TIACs of patients treated using rhTSH stimulation and THW, respectively. All statistical tests were exploratory and testing was performed at the two-sided 5% significance level. All statistical analysis was performed using GraphPad Prism version 9.3.1 or later for Windows (GraphPad Software, San Diego, California USA).

## Results

### Patient characteristics

One hundred and five patients were recruited at the four centres (Table 1). Twelve (11.4%), 1 (1.0%) and 92 (87.6%) patients received nominally 1.1, 2.5 and 3.7 GBq of Na[^131^I]I according to local protocols. All patients treated at centres 1 to 3 were administered 3.7 GBq, except for one patient receiving 2.5 GBq, while patients at centre 4 received either 1.1 or 3.7 GBq according to local standard-of-care. Of the 105 patients, 19 were treated under thyroid-hormone-withdrawal (THW) while the remaining patients had recombinant human thyroid-stimulating hormone (rhTSH) administered prior to treatment with Na[^131^I]I.

**Table 1:** Patient characteristics of the study participants at the four MEDIRAD WP3 centres

Characteristic	
Age—yr (mean ± standard deviation)	47.2 ± 15.6
Female—N (%) (*n* = 105)	79 (75.2)
Histological subtype—N (%)	
Papillary	87 (82.9)
Follicular	15 (14.3)
Mixed	3 (2.9)
Prescribed RAI activity—N (%)	
1100 MBq	12 (11.4)
2500 MBq	1 (1.0)
3700 MBq	92 (87.6)

### Dosimetry results

Dosimetry scans were collected for 37 single-time-point patients and 68 multiple-time-point patients for which two to six time-points between 6 and 168 h were available (see Table 2). Centres 1 to 3 performed two FOV SPECT scans covering the head/neck area to the lower abdomen, while centre 4 acquired a single FOV scan of the head/neck area.

**Table 2:** Summary of imaging data collected

	Centre 1 *n* = 34	Centre 2 *n* = 21	Centre 3 *n* = 25	Centre 4 *n* = 25
Single-time-point patients	None	None	25 (25 SPECT/CT, 1 per patient at 96 h)	12 (12 SPECT/CT, 1 per patient at 24 to 48 h)
Multiple-time-point patients (6 to 192 h)	34 (168 SPECT scans, 4 to 6 time-points per patient between 6 and 168 h)	21 (21 SPECT/CT and 77 SPECT scans, 4 to 6 time-points per patient between 6 and 168 h)	None	13 (13 SPECT/CT and 25 SPECT scans, 3 time-points per patient between 24 and 72 h except for 1 patient with only 2 scans)
Dosimetry performed by	DTB	DTA	DTA	DTA, (DTB for comparison of salivary glands only)

*DTA* dosimetry team A, *DTB* dosimetry team B

#### Normal organ absorbed doses

**Fig. 1:**
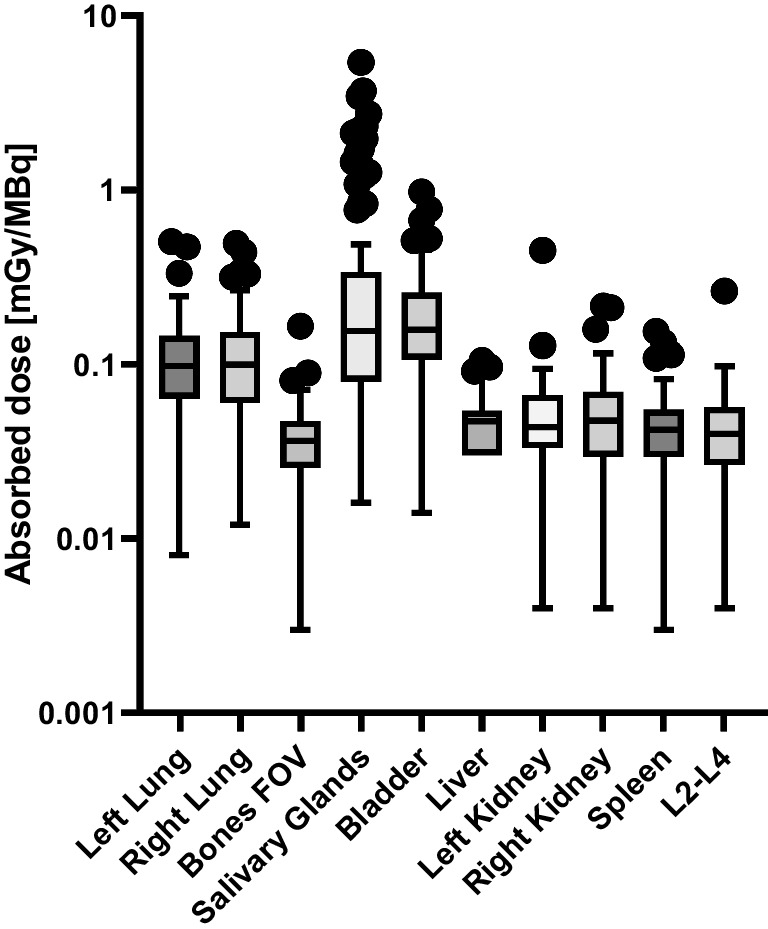
Ranges of absorbed dosed estimated for the patients (*n* = 105) in MEDIRAD WP3 for the lungs, bones, salivary glands, bladder, liver, kidneys, spleen and L2**–**L4. Results are shown for all four recruiting centres. Dosimetry for centre 1 was performed by DTB. Dosimetry for Centres 2, 3 and 4 was performed by dosimetry team A

Normal organ absorbed doses were estimated for the lungs, bones, salivary glands, bladder wall, liver, kidneys, spleen and L2–L4 (as a surrogate for the bone marrow absorbed dose). Absorbed doses per unit administered activity (mGy/MBq) are presented in Fig. 1 and summarised in Table 3. All dosimetry calculations presented here were performed by dosimetry team A except for those for centre 1 which were carried out by dosimetry team B.

**Table 3:** Median (range) of absorbed doses per administered activity (mGy/MBq) for the normal organs assessed for all patients combined and at the four different centres. Dosimetry for centre 1 was performed by dosimetry team B. Dosimetry for centres 2, 3 and 4 was performed by dosimetry team A

Organ	Centre 1 (mGy/MBq) *n* = 34	Centre 2 (mGy/MBq) *n* = 21	Centre 3 (mGy/MBq) *n* = 25	Centre 4 (mGy/MBq) *n* = 25
Left lung	-	0.1 (0.01–0.23)	0.08 (0.02–0.5)	0.11 (0.04–0.47)
Right lung	-	0.12 (0.01–0.44)	0.1 (0.03–0.33)	0.1 (0.04–0.49)
Bones	-	0.04 (0–0.07)	0.03 (0.01–0.16)	0.04 (0.02–0.08)
Salivary glands	0.44 (0.04–1.43)	0.14 (0.02–0.34)	0.05 (0.02–0.76)	0.16 (0.03–1.07)
Bladder wall	-	0.19 (0.01–0.97)	0.14 (0.02–0.66)	-
Liver	-	0.05 (0–0.11)	0.05 (0–0.09)	-
Left kidney	-	0.06 (0–0.13)	0.04 (0.01–0.45)	-
Right kidney	-	0.06 (0–0.21)	0.04 (0.01–0.21)	-
Spleen	-	0.06 (0–0.15)	0.04 (0.01–0.05)	-
L2–L4	-	0.05 (0–0.1)	0.03 (0.01–0.26)	-
Blood	-	0.08 (0.06–0.17)	-	-
Whole-body	0.04 (0.02–0.07)	0.05 (0.03–0.08)	0.04 (0.03–0.11)	0.04 (0.02–0.09)

**Fig. 2:**
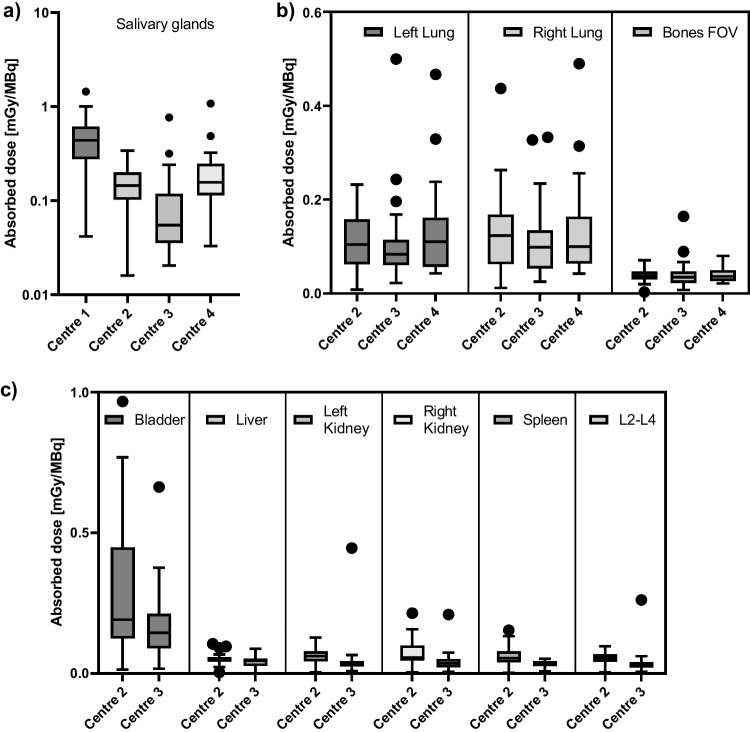
Range of absorbed doses per unit administered activity assessed for the **a** salivary glands, **b** lungs and bones and **c** bladder, liver, kidneys, spleen and L2–L4, respectively, presented for the individual centres (centre 1: *n* = 34, centre 2: *n* = 21, centre 3: *n* = 25 and centre 4: *n* = 25). Centre 1 had a SPECT-only system and only absorbed doses to the salivary glands could be determined, while centre 4 performed a single FOV scan which prevented quantification of any organs in the abdomen. Dosimetry calculations for centre 1 were performed by DTB. Absorbed doses for centres 2, 3 and 4 were calculated by dosimetry team A

Figure 2 shows the ranges of absorbed doses calculated for each of the centres individually. Ranges of absorbed doses delivered to the salivary glands, lungs and bones are comparable between centres 2 and 4. Salivary gland absorbed doses of centre 1, the centre with a SPECT-only system, are systematically higher, while salivary gland doses of centre 3, the centre with single-time-point imaging at 96 h, are lower. Ranges of absorbed doses for the bladder, liver, kidneys, spleen and L2–L4 could only be compared between centres 2 and 3 due to differences in the acquired FOV in centre 4, but a good agreement was found between centres 2 and 3.

#### Dosimetry comparison between dosimetry teams

**Fig. 3:**
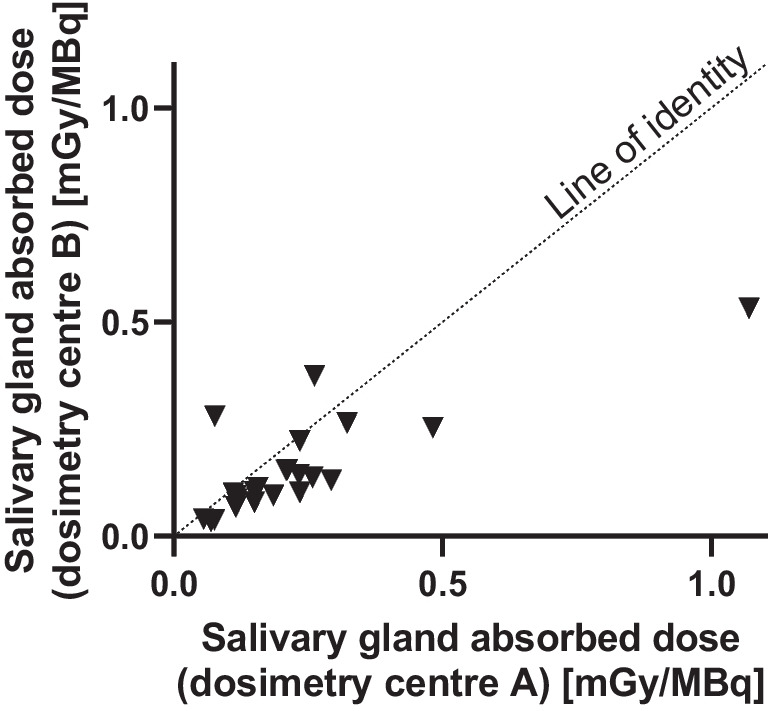
Comparison of salivary gland absorbed doses of the patients recruited at centre 4 (*n* = 25) between the two teams performing dosimetry

Salivary gland dosimetry results from the two dosimetry teams were compared for patients recruited at centre 4. Results are presented in Fig. 3. A good agreement was found between the results of both dosimetry teams.

#### Whole-body absorbed doses

**Fig. 4:**
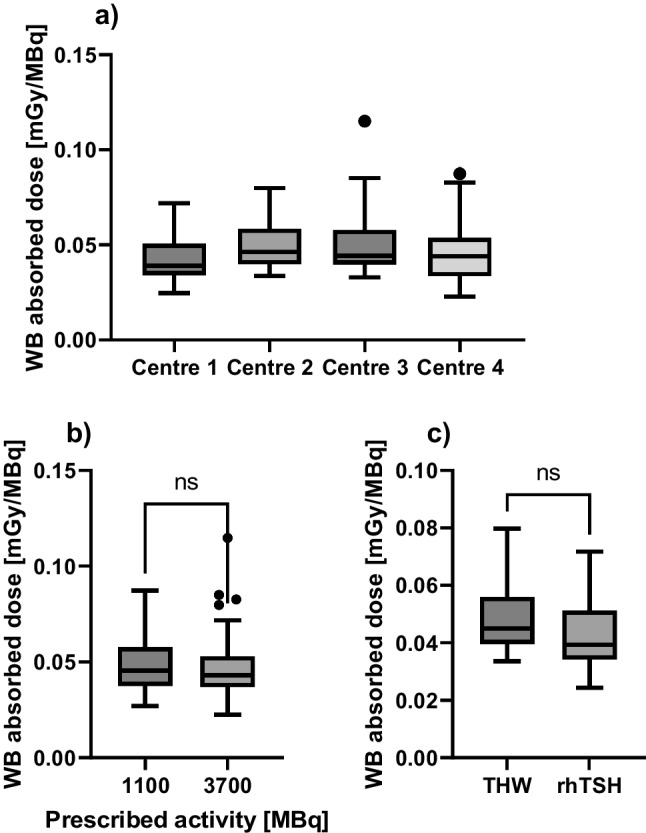
Comparison of the range of whole-body absorbed doses per unit administered (mGy/MBq) activity for **a** patients enrolled at each of the four study centres, **b** for patients treated with 1.1 GBq and 3.7 GBq and **c** for patients treated using THW and rhTSH (only for patients recruited in centres 1 and 2 due to the local differences in activity retention measurement protocols). The results of the Mann–Whitney test are indicated above each comparison with “ns” = non-significant (*p* value > 0.05)

Whole-body retention measurements were performed according to local protocols with median latest retention measurements at 167 h (range 45–174 h), 165 h (range 69–190 h), 42 h (range 30–112 h) and 44 h (range 19–70 h), respectively, for centres 1, 2, 3 and 4. Median TIACs for patients treated at centres 1, 2, 3 and 4 were 16.3 h (10.5–38.1 h), 20.0 h (14.4–34.8 h), 16.5 h (10.7–40.15 h) and 16.6 h (10.9–28.8 h), respectively. Figure 4a shows the comparison of whole-body absorbed doses per unit administered activity for the four recruiting centres which has also been added to Table 3. Figure 4b and c shows the comparison of whole-body absorbed doses per unit administered activity for patients treated with 1.1 and 3.7 GBq and between rhTSH stimulation and THW, respectively. As the time range of whole-body retention measurements was significantly different between centres 1 and 2 compared to centres 3 and 4, the comparison of rhTSH stimulation and THW was only performed for patients recruited in centres 1 and 2. Median WB absorbed doses per unit administered activity for patients treated using rhTSH stimulation and THW were 0.04 mSv/MBq (0.02–0.07 mSv/MBq) and 0.05 mSv/MBq (0.03–0.08 mSv/MBq), respectively. Interestingly, the difference in WB absorbed dose per unit administered activity between rhTSH stimulation and THW was found to be non-significant (*p* = 0.07) for patients treated in centres 1 and 2. Median TIACs for patients treated using rhTSH stimulation and THW were 16.3 h (10.5–38.1 h) and 19.7 h (14.4–28.0 h), respectively. The difference in TIACs for rhTSH and TWH patients was found to be significant (*p* = 0.02). The results of the Mann–Whitney test between the whole-body absorbed doses per unit administered activity for 1.1 and 3.7 GBq patients showed that the difference was non-significant (*p* = 0.60), indicating that whole-body absorbed doses scale with administered activity.

## Discussion

An important finding of this study is the large range of absorbed doses obtained for the normal organs, including the salivary glands and the bone marrow resulting from the administration of empirically-based fixed activity administrations of radioisotopes. This agrees with findings of previous studies [11, 30, 32]. Furthermore, whole-body absorbed doses appear to scale linearly with activity (see Fig. 4b) which is of significance when considering personalised treatment planning.

Dosimetry results reported here compare well to the literature. The median absorbed dose value per unit administered activity obtained in the present study of 0.15 mGy/MBq for the salivary glands is in agreement with the values of 0.2 mGy/MBq and 0.5 mGy/MBq provided by Jentzen et al. [36] for parotid and submandibular glands, respectively, and the ICRP publication 128 [37] estimate (blocked thyroid, oral administration model) of 0.26 mGy/MBq. Normal organ absorbed dose values for the lungs, liver, kidneys and spleen agree well with values reported by Kolbert et al. [38] for an rhTSH patient population and the respective ICRP publication 128 [37] estimates for healthy subjects with normal kidney function.

Whole-body absorbed doses were comparable between centres despite the variation in local practise of in-patient stays, and, therefore, the duration of activity retention measurements. Whole-body absorbed doses per unit administered activity were found to be not statistically significant different between rhTSH stimulation and THW. The large range of absorbed doses and differences in local acquisition protocols with respect to the whole-body retention measurements, which were performed according to local standard-of-care, may explain the difference to results presented by Hänscheid et al. [30]. THW was only used in a single centre in the present study and differences may be due to differences in local patient populations. Nevertheless, TIACs of rhTSH patients were found to be statistically significant lower when compared to THW patients, likely due to a reduction in the glomerular filtration rate in thyroid hormone withdrawal patients [39].

Salivary gland absorbed doses obtained from the centre with a SPECT-only system (centre 1) were found to be higher compared to other centres. The missing anatomical CT information, required for outlining and accurate attenuation correction, is a potential cause for these discrepancies. The comparison of dosimetry results by the two dosimetry teams for centre 4 suggests that discrepancies are not due differences in dosimetry methodologies but because of inaccurate quantification of salivary gland retention for centre 1. In addition, limited imaging protocols, such as the protocol in centre 3 with a single late imaging time-point at 96 h may prevent reasonable dosimetry estimates for example for the salivary glands. The latter have a relatively short effective half-life of approximately 9 h [32] which results in negligible physiological uptake at 96 h.

The development of personalised treatment approaches in MRT will require large-scale prospective studies which can only be performed in a multi-centre multi-national setting [40]. Multi-centre observational studies to collect absorbed doses in MRT, and the MEDIRAD study presented here, have shown that standardisation is challenging due to logistical differences and limitations in the ethical review process especially for observational studies. The results presented here indicate that data acquired in different centres may be collated even if flexible image acquisition protocols are implemented as ranges of absorbed doses are comparable. Several limitations on the flexibility of imaging schedules have been identified such as the lack of early imaging time-points for organs with short biological retention and lack of CT for accurate quantification. Further work is required to determine the level of standardisation and site set-up required for clinical trials depending on the specific trial endpoints [41].

Multi-centre observational studies will require suitably trained medical physics experts and a central dosimetry centre may be necessary for data processing to collate results from centres and investigate absorbed dose–response relationships in the case of non-standardised methodologies. Data processing in two dosimetry centres has proven to be very helpful to compare results and should be encouraged to promote exchange of dosimetry methodologies and tools while they are still under development. A limitation of the current study is that dosimetry was not compared for all patients between the two dosimetry teams.

## Conclusions

Multi-centre multi-national studies to assess absorbed doses to normal organs and target tissues are feasible in MRT. The results have shown that standardisation is not always achievable and required. Nevertheless, minimum standards might be required to achieve accurate quantification including the careful choice of imaging time-points and quantification methodologies. The large range of normal organ doses reported here shows the necessity for individualised dosimetry to allow recording and assessment of absorbed doses delivered during treatment. Further work is required to develop imaging networks and to evaluate the uncertainties associated with non-standardised acquisition protocols.

## Supplementary Information

Below is the link to the electronic supplementary material.

Supplementary file1 (PDF 465 kb)

## Data Availability

Data can be provided upon a reasonable request to the corresponding author.
