# Supplementary material for: Normal organ dosimetry for thyroid cancer patients treated with radioiodine as part of the multi-centre multi-national Horizon 2020 MEDIRAD project
Source: Eur J Nucl Med Mol Imaging. 2023 Jun 10;50(11):3225–34. doi: 10.1007/s00259-023-06295-0 (PMC10256579; doi:10.1007/s00259-023-06295-0)
Supplement: Supplementary file 1 — Supplementary file1 (PDF 465 kb) [file 259_2023_6295_MOESM1_ESM.pdf]

Supplementary material:

Taprogge et al: Normal organ dosimetry for thyroid cancer patients treated with radioiodine as part of the multi-centre multi-national MEDIRAD project

Supplementary Table 1: Ethics approval WP3

Investigating centre	*Ethical approval obtained*	*Ethics approval details*	*National, EudraCT or ClinicalTrials.gov Identifier*
UKW	9th May 2019	Approved by the National Ethics Committee	EudraCT: 2019-002244-25
UMR	14th May 2019	Approved by the National Ethics Committee	EudraCT: 2019-002244-25
IUCT-O	16th Dec 2019	Approved by the National Ethics Committee	ID RCB : 2019-A01734-53
RMH/ICR	19th March 2020	The study was approved by the East Midlands - Nottingham 1 Research Ethics Committee (20/EM/0022) and the institutional review board at the Royal Marsden Hospital	ClinicalTrials.gov: NCT04391244

Supplementary Table 2: Acquisition parameters used for ^{131}I imaging as part of the MEDIRAD WP3 study.

	^{131}I acquisition protocol
Collimator	High Energy
Photopeak energy window	364 keV ± 10% or ± 15%
SPECT(/CT) Matrix	128 x 128
SPECT movement	Body contour
Projections	2 x 30 (6° projection) or 2 x 36 (5° projection)
Time per projection	Adjusted based on measured count-rate for patient acquisition
CT	Standard low-dose protocol (if applicable)

Supplementary Table 3: SPECT (/CT) reconstruction parameters used for ^{131}I imaging as part of the MEDIRAD WP3 study.

	^{131}I *reconstruction protocol*
Reconstruction	OSEM (4 iterations, 10 subsets)
Attenuation correction (AC)	CTAC (One centre: Chang with 0.11 cm^{-1} @ 364 keV)
Scatter correction	Triple-Energy Window (TEW)
Post-reconstruction filtering	None
